# MPH: fast REML for large-scale genome partitioning of quantitative genetic variation

**DOI:** 10.1093/bioinformatics/btae298

**Published:** 2024-04-30

**Authors:** Jicai Jiang

**Affiliations:** Department of Animal Science, North Carolina State University, Raleigh, NC 27695, United States

## Abstract

**Motivation:**

Genome partitioning of quantitative genetic variation is useful for dissecting the genetic architecture of complex traits. However, existing methods, such as Haseman–Elston regression and linkage disequilibrium score regression, often face limitations when handling extensive farm animal datasets, as demonstrated in this study.

**Results:**

To overcome this challenge, we present MPH, a novel software tool designed for efficient genome partitioning analyses using restricted maximum likelihood. The computational efficiency of MPH primarily stems from two key factors: the utilization of stochastic trace estimators and the comprehensive implementation of parallel computation. Evaluations with simulated and real datasets demonstrate that MPH achieves comparable accuracy and significantly enhances convergence, speed, and memory efficiency compared to widely used tools like GCTA and LDAK. These advancements facilitate large-scale, comprehensive analyses of complex genetic architectures in farm animals.

**Availability and implementation:**

The MPH software is available at https://jiang18.github.io/mph/.

## 1 Introduction

Genomic partitioning is a method for investigating the genetic architecture of complex traits by grouping whole-genome SNPs in a predetermined manner and estimating their contributions to genetic variation (Yang *et al.* 2011b). Early work on genome partitioning has used genomic restricted maximum likelihood (REML) based on a variety of features, e.g. biological pathways (Edwards *et al.* 2015), chromosomes (Pimentel *et al.* 2011), minor allele frequency (MAF) and linkage disequilibrium (LD) (Yang *et al.* 2015), or non-overlapping SNP annotations (Gusev *et al.* 2014). All these studies enhanced our understanding of the genetic architecture of complex traits. Still, they were performed with a small number of variance components (VCs) and/or a relatively small sample. Efficient REML-based tools to analyze many VCs for large-scale genotype data are sorely lacking. Though some REML implementations such as GCTA (Yang *et al.* 2011a) and LDAK (Speed *et al.* 2012) have been well optimized, using them for many VCs often becomes computationally intractable at sample sizes in the tens of thousands. A Monte Carlo method has been employed to speed up REML as implemented in BOLT (Loh *et al.* 2015). It can analyze biobank-scale samples; however, it becomes slow when modeling millions of sequence variants. In addition, BOLT currently cannot partition genetic variance by overlapping SNP categories that are common when considering multiple functional annotations.

Finucane *et al.* (2015) proposed stratified linkage disequilibrium score regression (S-LDSC or simply LDSC) to tackle these problems in REML analyses. LDSC approximates REML, and it requires GWAS summary statistics and LD information rather than individual genotypes and phenotypes. LDSC can simultaneously estimate many VCs, facilitating the incorporation of functional annotations into human genetic studies (Finucane *et al.* 2018, Marquez-Luna *et al.* 2021). Several other methods have also been developed for the same purpose, e.g. RHE-mc-reg (Pazokitoroudi *et al.* 2020), MQS (Zhou 2017), and SumHer (Speed and Balding 2019). It is appealing to do similar analyses in farm animals, as they can help to understand the genetic basis of animal productivity traits and potentially improve genomic predictions. Unfortunately, current methods predominantly rely on approximations tailored to human datasets characterized by weak LD and limited relatedness among individuals. As demonstrated theoretically and empirically in this study, these methods are suboptimal for livestock data, where substantial relatedness and strong LD are prevalent. The lack of LDSC-like tools for farm animals hinders the use of the increasing amount of functional annotation data generated by the Functional Annotation of ANimal Genomes (FAANG) and related projects (Giuffra *et al.* 2019, Chamberlain *et al.* 2021).

To tackle this issue, we present a new REML software tool for partitioning quantitative genetic variation in a framework of linear mixed models, which we refer to as MPH. We apply MPH to various simulated and real datasets, demonstrating its enhanced convergence, speed, and memory efficiency for enabling large-scale, complex genome partitioning.

## 2 Methods

### 2.1 LD score regression and Haseman–Elston regression

Many fast methods have been developed to estimate VCs for complex traits in human populations. We here use two typical ones, LDSC (Bulik-Sullivan *et al.* 2015) and Haseman–Elston regression (HE-reg) (Haseman and Elston 1972), to illustrate that they use approximations tailored to human populations and cannot be applied to livestock data. For simplicity, we focus on a model with only one genetic component, but our derivation holds for multi-component models.

Given covariate-adjusted phenotypes (**y**) of *N* individuals and standardized genotypes (**Z**) of *M* SNPs, we have y=Zα+e, where SNP effects $\alpha ∼N(0,I\sigma_{\alpha }^{2})$ and residuals $e∼N(0,I\sigma_{e}^{2})$. A simple transformation gives the following model:

(1) $$\frac{Z^{T}y}{\sqrt{N\sigma_{p}^{2}}}∼N\left(0,\frac{Z^{T}ZZ^{T}Z\sigma_{\alpha }^{2}}{N\sigma_{p}^{2}}+\frac{Z^{T}Z\sigma_{e}^{2}}{N\sigma_{p}^{2}}\right),$$

where $\sigma_{p}^{2}$ is the phenotypic variance. Note that $Z^{T}y/\sqrt{N\sigma_{p}^{2}}$ and $Z^{T}Z/N$ are linear regression *z*-scores (denoted as **s**) for single-SNP associations and the genotype correlation matrix (denoted as **C**), respectively (we ignore the error introduced by the use of *N* instead of *N−*1). Model (1) becomes

(2) $$s∼N\left(0,NC^{2}h^{2}/M+C\left(1-h^{2}\right)\right),$$

where *h*^2^ is SNP heritability (${{M\sigma }_{\alpha }^{2}=h^{2}\sigma }_{p}^{2}$). Model (2) does not simplify *h*^2^ estimation, because its dimension *M* is usually very large, reaching millions for sequence genotypes. LDSC further approximates this model by

(3) $$s∼N\left(0,\mathrm{diag}\left(NC^{2}h^{2}/M+C\left(1-h^{2}\right)\right)\right);$$

that is, the covariance matrix is approximated by its diagonal. From model (3), we can readily obtain for SNP *j*

(4) $$s_{j}∼N\left(0,N\ell_{j}h^{2}/M+\left(1-h^{2}\right)\right),$$

where *ℓ_j_* is the *j*th diagonal element of **C**^2^. It is computed as the sum of the squared correlations between SNP *j* and all SNPs genome-wide, commonly referred to as LD score (Bulik-Sullivan *et al.* 2015). The method of iteratively reweighted least squares can subsequently be used to obtain the maximum likelihood estimate of *h*^2^. It is important to note that LDSC considers the estimation of *ℓ_j_* with a reference population and the potential presence of confounding factors, so model (4) is not precisely the model upon which LDSC is based. However, LDSC still relies on the approximation of model (2) by (3). The effectiveness of this approximation depends on the rapid decay of LD over distance, leading to predominantly negligible off-diagonal elements in **C**. Consequently, the LDSC framework is not suitable for livestock genomes, which are characterized by prevalent strong, long-span LDs.

Let $G=ZZ^{T}/M$ and $\sigma_{g}^{2}=M\sigma_{\alpha }^{2}$. HE-reg uses a method-of-moments estimator satisfying the following normal equations:

(5) $$\left[\begin{array}{cc} tr(\mathrm{GG}) & tr(G)\\ tr(G) & N \end{array}\right]\left[\begin{array}{c} \hat{\sigma_{g}^{2}}\\ \hat{\sigma_{e}^{2}} \end{array}\right]=\left[\begin{array}{c} y^{T}\mathrm{Gy}\\ y^{T}y \end{array}\right].$$

It can be revealed that the HE-reg equations represent a special case of the MINQUE (minimum norm quadratic unbiased estimation) equations as shown below (Rao 1971)

(6) $$\left[\begin{array}{cc} tr({\tilde{V}}^{-1}G{\tilde{V}}^{-1}G) & tr({\tilde{V}}^{-1}G{\tilde{V}}^{-1})\\ tr({\tilde{V}}^{-1}{\tilde{V}}^{-1}G) & tr({\tilde{V}}^{-1}{\tilde{V}}^{-1}) \end{array}\right]\left[\begin{array}{c} \hat{\sigma_{g}^{2}}\\ \hat{\sigma_{e}^{2}} \end{array}\right]=\left[\begin{array}{c} y^{T}{\tilde{V}}^{-1}G{\tilde{V}}^{-1}y\\ y^{T}{\tilde{V}}^{-1}{\tilde{V}}^{-1}y \end{array}\right],$$

where $\tilde{V}=G\tilde{\sigma_{g}^{2}}+I\tilde{\sigma_{e}^{2}}$. $\tilde{\sigma_{g}^{2}}$ and $\tilde{\sigma_{e}^{2}}$, serving as *a priori* values within $\tilde{V}$, influence the statistical efficiency of MINQUE. Specifically, MINQUE achieves optimal efficiency when the ratio of *a priori* values matches the ratio of true VCs. MINQUE equations (6) reduce to HE-reg equations (5) when $\tilde{V}$ is set to a product of a constant scalar and an identity matrix. This $\tilde{V}$ setting is close to optimal only when $\sigma_{g}^{2}$ is small relative to $\sigma_{e}^{2}$ or when **G** is approximately an identity matrix. In other words, HE-reg is effective only when trait heritability approaches zero or when the individuals under analysis are largely unrelated. In brief, the HE-reg method is not well suited for livestock samples characterized by overall substantial relatedness among individuals.

In summary, LDSC, HE-reg, and analogous methods are primarily based on approximations tailored to human populations. Few of these methods can produce statistically efficient estimates for animal genomic studies, where individuals are often related and exhibit strong LD across the genome. This limitation is further corroborated by simulation analyses using cattle and pig genotype data (see Section 2.4 for details).

### 2.2 Linear mixed model

MPH fits the following multi-component linear mixed model:

(7) $$\begin{array}{c} y=X\beta +g+e\\ g∼N(0,\sum_{k=1}^{K}G_{k}\eta_{k}),\\ e∼N(0,R\sigma_{e}^{2}) \end{array}$$

where **y** is a vector of phenotypes, **β** is a vector of fixed effects, **X** is the design matrix of **β**, **g** is a vector of random effects with *K* VCs, **G**_*k*_ ($k\in \left\{1,\ldots ,K\right\}$) is the *k*th precomputed covariance structure matrix corresponding to VC $\eta_{k}$, and **e** is the residual with diagonal covariance structure matrix **R** and VC $\sigma_{e}^{2}$. In the genome partitioning analysis, **G**_*k*_ is usually a genomic relationship matrix (GRM). Assuming that **g** is independent of **e**, the total variance–covariance matrix is as follows:

$$V=\sum_{k=1}^{K}G_{k}\eta_{k}+R\sigma_{e}^{2}.$$

The VC parameter space is defined as all values of VCs that result in positive definite **V**.

Model (7) is general enough for genome partitioning of quantitative genetic variation; for example, if we want to analyze both genetic effects and permanent environmental effects, we can consider their sum by $g=g_{G}+g_{\mathrm{PE}}$ in which $g_{G}$ is a vector of genetic effects and $g_{\mathrm{PE}}$ is a vector of permanent environmental effects. The variance–covariance matrix of **g** is therefore $\mathrm{Var}\left(g\right)=\mathrm{Var}\left(g_{G}\right)+\mathrm{Var}\left(g_{\mathrm{PE}}\right)+2C\mathrm{ov}(g_{G},g_{\mathrm{PE}})$, and it is easy to obtain a form of $\mathrm{Var}\left(g\right)$ as in model (7).

### 2.3 Algorithms and software

MPH integrates several algorithms to facilitate fast REML estimation of VCs. First, the REML estimates are computed using Fisher’s scoring method, and their corresponding analytical standard errors are derived from the Fisher information matrix. Second, the trust-region dogleg method (Yuan 2000) is implemented to overcome possible convergence failures in REML resulting from non-positive definite **V**. Such failures tend to occur more often than usual in complex genomic partitioning. Third, MPH utilizes a stochastic trace estimator (Hutchinson 1989) to accelerate trace term evaluations in REML, contrasting with direct computations conventionally employed by software like GCTA and LDAK. As a result, MPH may not yield identical VC estimates as conventional Fisher-scoring REML methods. Details on the algorithms are described in the Supplementary Notes.

MPH is written in C++ and utilizes the Eigen library. Its memory usage is optimized, particularly through packed storage of symmetric GRMs and on-the-fly GRM reading. MPH’s REML requires ∼8 *N*^2^ bytes of memory when GRMs are read on the fly and (2*K *+* *4)*N*^2^ bytes otherwise, with *N* representing the number of individuals. Its theoretical time complexity is *O*(*N*^3^+*KN*^2^), with the dominant factor being the Cholesky decomposition of **V**, which is efficiently performed using the Eigen library.

The computational efficiency of MPH primarily stems from two key factors: the utilization of stochastic trace estimators and the implementation of parallel computation. Specifically, parallelization is applied not only to matrix operations but also to the reading of GRMs.

### 2.4 Data and benchmarking

Regarding computational efficiency, convergence, and statistical efficiency, we compared MPH against judiciously selected counterparts, including conventional REML implementations in LDAK and GCTA, the Monte Carlo REML implementation in BOLT, HE-reg in LDAK, and two summary-statistics-based methods in MQS (-HEW and -LDW). Specifically, we considered LDAK’s average-information (AI) REML and GCTA’s AI and Fisher-scoring REML algorithms for comparison. MQS-HEW is mathematically equivalent to HE-reg, whereas MQS-LDW offers an exact estimation form of LDSC (Zhou 2017). Not all counterparts were included in every benchmarking analysis, as deemed unnecessary; for instance, HE-reg and MQS were excluded from computation resource comparisons due to their poor statistical efficiency.

We simulated a genotype dataset of 50k unrelated animals with 5M sequence variants (MAF ≥ 1%) evenly distributed on 30 chromosomes using the software genosim (VanRaden *et al.* 2015). Subsequently, we simulated phenotypes for a single trait with a heritability of 0.5, assuming an equal contribution from all chromosomes. We used this trait to compare the speed and memory usage of MPH, GCTA, LDAK, and BOLT in the REML analysis of partitioning SNP heritability across 30 chromosomes. Additionally, we sampled 10k individuals and simulated phenotypes for 100 independent traits, each with a heritability of 0.5, under three scenarios: (1) a VC of 1 for each of chromosomes 1–15 and 0 for each of chromosomes 16–30, (2) 1 for all chromosomes, and (3) 1 for each of chromosomes 1–15 and 2 for each of chromosomes 16–30. We then utilized these replicates (3 × 100) to compare the REML convergence of MPH and LDAK for partitioning SNP heritability across chromosomes.

We accessed the accuracy of MPH’s REML using two publicly available real animal datasets: dairy bull (Zhang *et al.* 2015) and Duroc pig (Yang *et al.* 2021). We preprocessed each dataset (e.g. quality control), as described by Cheng *et al.* (2023). In our assessment, we randomly split SNPs in each dataset into two equal sets and assigned them genetic VC values of 1 and 4 to simulate phenotypes for 100 independent traits, each with a heritability of 0.5. We then used these simulated phenotypes to compare the VC estimates of MPH, LDAK’s REML and HE-reg, GCTA’s Fisher-scoring REML, and MQS (-HEW and -LDW). In particular, the entire individual-level data used for GWAS was set as the reference for MQS in this benchmarking analysis. Additionally, we used the real phenotypes to compare MPH to LDAK and GCTA’s REMLs.

## 3 Results

### 3.1 Computational speed and memory usage

**Figure 1.**
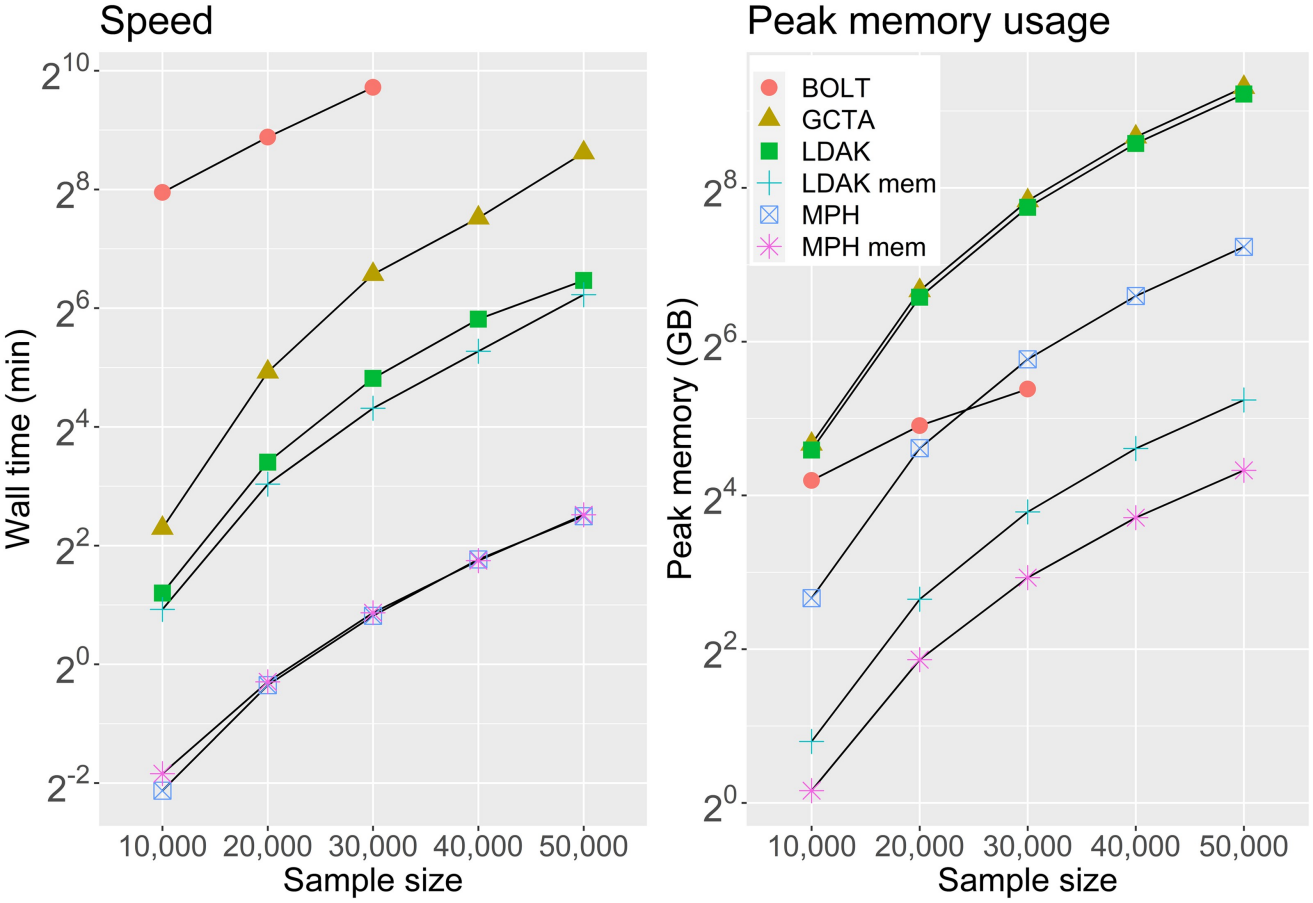
Computational speed and peak memory usage of MPH, GCTA, LDAK, and BOLT in REML for partitioning SNP heritability across 30 chromosomes. The *y*-axis is on a logarithmic scale. MPH mem and LDAK mem indicate their memory-saving modes. All the software tools utilized 10 Intel Xeon Gold 6230 CPU cores. GCTA, LDAK, and BOLT used AI REML in this analysis. The wall time for GCTA, LDAK, and MPH is attributed to their --reml options.

As shown in Fig. 1, MPH’s REML was overall over 10 times faster than LDAK’s, which was, in turn, 2–3 times faster than GCTA’s. BOLT was significantly slower than its counterparts when handling 5 million sequence variants in its REML analysis. The memory-saving modes of MPH and LDAK, which read GRMs on the fly, did not reduce computational speed, likely due to file caching. Interestingly, LDAK’s memory-saving mode even showed increased speed. When the memory-saving mode was disabled, MPH utilized ∼1/4 of memory compared to GCTA and LDAK. MPH’s memory-saving mode used half as much memory as LDAK’s.

MPH’s memory-saving REML exhibited a practical time complexity of approximately *O*(*KN*^2^), where *K* represents the number of VCs and *N* represents the sample size (Supplementary Fig. S1). In particular, it took 26.6 min and utilized 59 GB of peak memory to partition SNP heritability across 30 chromosomes in a sample of 100k individuals when using 14 Intel Xeon Gold 6258R CPU cores (Supplementary Fig. S1).

### 3.2 Convergence

Table 1 displays the REML convergence performance of MPH and LDAK for partitioning SNP heritability across 30 chromosomes in a sample of 10k unrelated individuals. MPH failed to converge in 3 out of 100 replicates under Scenario 1 (VC = 1 for each of chromosomes 1–15 and VC = 0 for each of 16–30) but succeeded in all replicates under Scenarios 2–3. In contrast, LDAK with HE starts (using HE-reg estimates as starting values for REML) had convergence issues in 56, 1, and 2 out of 100 replicates under Scenarios 1–3, respectively. Without HE starts, LDAK’s performance was notably poorer. All three failures of MPH were associated with an exploding gradient for a simulated VC of 0. Excluding the respective chromosome resolved the REML convergence issue in these cases. In addition, as expected, convergence failures led to inaccurate VC estimates (Supplementary Fig. S2).

**Table 1.** Number of REML convergence failures out of 100 replicates for partitioning heritability across 30 chromosomes by MPH and LDAK in a sample of 10k unrelated individuals under three scenarios.

Scenario	True VC values		MPH	LDAK REML	
	Chr1–15	Chr16–30		HE starts	Naïve starts
1	1	0	3	56	98
2	1	1	0	1	2
3	1	2	0	2	26

The initial VC values for MPH were set to match LDAK’s naïve starts, establishing equal initial values for the VCs across 30 chromosomes with a heritability of 0.5. In the context of HE starts, LDAK initially performs HE-reg and then uses the resulting estimates as initial VC values for REML.

### 3.3 Accuracy and statistical efficiency

The simulation analysis, using real genotypes of dairy bulls and Duroc pigs, demonstrated that MPH (Fisher-scoring REML), LDAK (AI REML), and GCTA (Fisher-scoring REML) produced practically identical VC estimates, despite differences in their REML methods (Supplementary Fig. S3). MPH and GCTA, both utilizing Fisher-scoring REML, yielded similar analytical standard errors (SEs) of VC estimates, while LDAK’s AI REML exhibited slightly different SEs (Supplementary Fig. S4). The results from the simulation analysis largely mirrored those for the real phenotypes of dairy bulls and Duroc pigs (Supplementary Tables S1 and S2).

Consistent with the theoretical analysis in Section 2.1, HE-reg, MQS-HEW (equivalent to HE-reg), and MQS-LDW (analogous to LDSC) showed poor statistical efficiency compared to REML for simulated traits based on real genotypes of dairy bulls and Duroc pigs (Supplementary Fig. S5). Furthermore, increasing sample size led to a substantially weaker gain in statistical power for HE-reg, MQS-HEW, and MQS-LDW than for REML (Supplementary Fig. S6). It is noteworthy that the LD scores used for the MQS-LDW -wcat option in this benchmarking analysis were calculated as the sum of observed squared correlations between SNPs, but using expected squared correlations (equal to observed values minus 1/*N*) for LD scores led to similar VC estimates (Supplementary Fig. S7).

## 4 Discussion

Our theoretical and empirical analyses demonstrate that LDSC, HE-reg, and analogous methods lack statistical efficiency for genomic studies in animal populations. To address the need for large-scale genome partitioning of quantitative genetic variation in farm animal traits, we developed MPH, a new REML software tool. As demonstrated by data analyses, MPH offers comparable accuracy and significant improvements in convergence, speed, and memory efficiency compared to widely used tools such as GCTA and LDAK.

MPH is particularly well-suited for analyses involving a large number (e.g. 10M) of (imputed) sequence variants, a moderate sample size (10k–100k), and numerous (e.g. 50) VCs. However, it should be noted that other REML tools may outperform MPH in certain analyses. For example, BOLT excels in partitioning genetic variance based on disjoint sets of a limited number (e.g. 100k) of genomic variants, while SLEMM represents a suitable alternative when estimating only one genetic component (Cheng *et al.* 2023). It is important to acknowledge that the current scope of MPH is limited to single-variate REML analyses. Future research will focus on extending and evaluating MPH for bivariate and multivariate analyses.

In summary, MPH represents a useful tool for researchers studying the genetic architecture of complex traits, providing high performance in a variety of large-scale REML-based genetic analyses. Access to the source code, pre-compiled binary, documentation, and multiple example analyses is available at https://jiang18.github.io/mph.

## Supplementary Material

btae298_Supplementary_Data

## Data Availability

The dairy bull and Duroc pig datasets used to demonstrate our method were obtained from https://doi.org/10.1534/g3.114.016261 and https://doi.org/10.5524/100894, respectively.
