# Supplementary material for: MPH: fast REML for large-scale genome partitioning of quantitative genetic variation
Source: Bioinformatics. 2024 Apr 30;40(5):btae298. doi: 10.1093/bioinformatics/btae298 (PMC11093526; doi:10.1093/bioinformatics/btae298)
Supplement: btae298_Supplementary_Data [file btae298_supplementary_data.pdf]

Supplementary data

MPH: fast REML for large-scale genome partitioning of quantitative genetic variation

Jicai Jiang

Supplementary Notes

1. Variance component estimation

1.1. Minimum norm quadratic unbiased estimation (MINQUE) ……………………..2

1.2. Restricted maximum likelihood (REML) ……………………………………….2

1.3. Trust-region dogleg method ……………………………………………………..4

1.4. Stochastic trace estimator ....…...5

2. Phenotype simulation …………………………………………………………………6

Supplementary Figures S1 to S7

Supplementary Tables S1 and S2

References

Supplementary Notes

1. Variance component estimation

1.1. Minimum norm quadratic unbiased estimation (MINQUE)

The K+1 variance components (VCs) in model (7) of the main text can be estimated by the following MINQUE equations (Rao, 1971; Rao, 1972):

$$\begin{bmatrix} \mathrm{tr}\left(\tilde{\mathbf{P}}\mathbf{G}_1\tilde{\mathbf{P}}\mathbf{G}_1\right) & \cdots & \mathrm{tr}\left(\tilde{\mathbf{P}}\mathbf{G}_1\tilde{\mathbf{P}}\mathbf{G}_K\right) & \mathrm{tr}\left(\tilde{\mathbf{P}}\mathbf{G}_1\tilde{\mathbf{P}}\mathbf{R}\right) \\ \vdots & \ddots & \vdots & \vdots \\ \mathrm{tr}\left(\tilde{\mathbf{P}}\mathbf{G}_K\tilde{\mathbf{P}}\mathbf{G}_1\right) & \cdots & \mathrm{tr}\left(\tilde{\mathbf{P}}\mathbf{G}_K\tilde{\mathbf{P}}\mathbf{G}_K\right) & \mathrm{tr}\left(\tilde{\mathbf{P}}\mathbf{G}_K\tilde{\mathbf{P}}\mathbf{R}\right) \\ \mathrm{tr}\left(\tilde{\mathbf{P}}\mathbf{R}\tilde{\mathbf{P}}\mathbf{G}_1\right) & \cdots & \mathrm{tr}\left(\tilde{\mathbf{P}}\mathbf{R}\tilde{\mathbf{P}}\mathbf{G}_K\right) & \mathrm{tr}\left(\tilde{\mathbf{P}}\mathbf{R}\tilde{\mathbf{P}}\mathbf{R}\right) \end{bmatrix} \begin{bmatrix} \eta_1 \\ \vdots \\ \eta_K \\ \sigma_e^2 \end{bmatrix} = \begin{bmatrix} \mathbf{y}^{\mathrm{T}}\tilde{\mathbf{P}}\mathbf{G}_1\tilde{\mathbf{P}}\mathbf{y} \\ \vdots \\ \mathbf{y}^{\mathrm{T}}\tilde{\mathbf{P}}\mathbf{G}_K\tilde{\mathbf{P}}\mathbf{y} \\ \mathbf{y}^{\mathrm{T}}\tilde{\mathbf{P}}\mathbf{R}\tilde{\mathbf{P}}\mathbf{y} \end{bmatrix}, \qquad \text{(A1)}$$

where $\tilde{\mathbf{P}} = \tilde{\mathbf{V}}^{-1} - \tilde{\mathbf{V}}^{-1}\mathbf{X}\left(\mathbf{X}^{\mathrm{T}}\tilde{\mathbf{V}}^{-1}\mathbf{X}\right)^{-1}\mathbf{X}^{\mathrm{T}}\tilde{\mathbf{V}}^{-1}$ and $\tilde{\mathbf{V}} = \sum_k \mathbf{G}_k\widetilde{\eta_k} + \mathbf{R}\widetilde{\sigma_e^2}$. $\widetilde{\eta_k}$ ($k \in \{1, \cdots, K\}$) and $\widetilde{\sigma_e^2}$ are a priori values for VCs, and as long as they do not depend on the data, MINQUE is unbiased. The statistical efficiency of MINQUE is influenced by the a priori values of VCs. Several choices of VC prior values are often used in MINQUE. If zero prior values are assigned to all VCs except the residual variance, the scheme is called MINQUE(0). When $\mathbf{R}$ is identity, MINQUE(0) becomes the Haseman-Elston regression (HE-reg) estimator used by Pazokitoroudi, et al. (2020). If all prior values are set to 1, the scheme is called MINQUE(1). MINQUE does not depend on the normality of the variables [$\mathbf{y}$, $\mathbf{g}$, and $\mathbf{e}$ in model (7) of the main text]. When these variables are normally distributed, the MINQUE equations coincide with those of minimum variance quadratic unbiased estimation (MIVQUE) (Rao, 1971).

In MINQUE(0) and MINQUE(1), equation (A1) is solved once to obtain the VC estimates. An iterative estimation procedure, known as iterative MINQUE (I-MINQUE), can be constructed using current VC estimates as prior values for subsequent iterations. Brown (1976) demonstrated that I-MINQUE is asymptotically efficient when the variables are assumed to be normally distributed. Under normality, I-MINQUE is equivalent to restricted maximum likelihood (REML) (Swallow and Monahan, 1984).

1.2. Restricted maximum likelihood (REML)

The REML log-likelihood function for model (7) of the main text is

$$\ell\left(\eta_1, \cdots, \eta_K, \sigma_e^2\right) = -\frac{n-c}{2}\log\left(2\pi\right) + \frac{1}{2}\log\left|\mathbf{X}^{\mathrm{T}}\mathbf{X}\right| - \frac{1}{2}\log\left|\mathbf{V}\right| - \frac{1}{2}\log\left|\mathbf{X}^{\mathrm{T}}\mathbf{V}^{-1}\mathbf{X}\right| - \frac{1}{2}\mathbf{y}^{\mathrm{T}}\mathbf{P}\mathbf{y}, \qquad \text{(A2)}$$

where $\mathbf{V} = \sum_k \mathbf{G}_k\eta_k + \mathbf{R}\sigma_e^2$ and $\mathbf{P} = \mathbf{V}^{-1} - \mathbf{V}^{-1}\mathbf{X}(\mathbf{X}^{\mathrm{T}}\mathbf{V}^{-1}\mathbf{X})^{-1}\mathbf{X}^{\mathrm{T}}\mathbf{V}^{-1}$. The gradient vector and Hessian matrix of function (A2) are therefore

$$\nabla \ell = \begin{bmatrix} \partial\ell/\partial\eta_1 \\ \vdots \\ \partial\ell/\partial\eta_K \\ \partial\ell/\partial\sigma_e^2 \end{bmatrix} = -\frac{1}{2}\begin{bmatrix} \mathrm{tr}(\mathbf{PG}_1) - \mathbf{y}^{\mathrm{T}}\mathbf{PG}_1\mathbf{Py} \\ \vdots \\ \mathrm{tr}(\mathbf{PG}_K) - \mathbf{y}^{\mathrm{T}}\mathbf{PG}_K\mathbf{Py} \\ \mathrm{tr}(\mathbf{PR}) - \mathbf{y}^{\mathrm{T}}\mathbf{PRPy} \end{bmatrix} \tag{A3}$$

and

$$\nabla^2 \ell = -\frac{1}{2}\begin{bmatrix} 2\mathbf{y}^{\mathrm{T}}\mathbf{PG}_1\mathbf{PG}_1\mathbf{Py} - \mathrm{tr}(\mathbf{PG}_1\mathbf{PG}_1) & \cdots & 2\mathbf{y}^{\mathrm{T}}\mathbf{PG}_1\mathbf{PG}_K\mathbf{Py} - \mathrm{tr}(\mathbf{PG}_1\mathbf{PG}_K) & 2\mathbf{y}^{\mathrm{T}}\mathbf{PG}_1\mathbf{PRPy} - \mathrm{tr}(\mathbf{PG}_1\mathbf{PR}) \\ \vdots & \ddots & \vdots & \vdots \\ 2\mathbf{y}^{\mathrm{T}}\mathbf{PG}_K\mathbf{PG}_1\mathbf{Py} - \mathrm{tr}(\mathbf{PG}_K\mathbf{PG}_1) & \cdots & 2\mathbf{y}^{\mathrm{T}}\mathbf{PG}_K\mathbf{PG}_K\mathbf{Py} - \mathrm{tr}(\mathbf{PG}_K\mathbf{PG}_K) & 2\mathbf{y}^{\mathrm{T}}\mathbf{PG}_K\mathbf{PRPy} - \mathrm{tr}(\mathbf{PG}_K\mathbf{PR}) \\ 2\mathbf{y}^{\mathrm{T}}\mathbf{PRPG}_1\mathbf{Py} - \mathrm{tr}(\mathbf{PRPG}_1) & \cdots & 2\mathbf{y}^{\mathrm{T}}\mathbf{PRPG}_K\mathbf{Py} - \mathrm{tr}(\mathbf{PRPG}_K) & 2\mathbf{y}^{\mathrm{T}}\mathbf{PRPRPy} - \mathrm{tr}(\mathbf{PRPR}) \end{bmatrix}, \tag{A4}$$

respectively, where tr() represents the trace of a matrix. Following equation (A4), we can derive the Fisher information (FI) matrix

$$\mathrm{FI}(\eta_1, \cdots, \eta_K, \sigma_e^2) = \frac{1}{2}\begin{bmatrix} \mathrm{tr}(\mathbf{PG}_1\mathbf{PG}_1) & \cdots & \mathrm{tr}(\mathbf{PG}_1\mathbf{PG}_K) & \mathrm{tr}(\mathbf{PG}_1\mathbf{PR}) \\ \vdots & \ddots & \vdots & \vdots \\ \mathrm{tr}(\mathbf{PG}_K\mathbf{PG}_1) & \cdots & \mathrm{tr}(\mathbf{PG}_K\mathbf{PG}_K) & \mathrm{tr}(\mathbf{PG}_K\mathbf{PR}) \\ \mathrm{tr}(\mathbf{PRPG}_1) & \cdots & \mathrm{tr}(\mathbf{PRPG}_K) & \mathrm{tr}(\mathbf{PRPR}) \end{bmatrix} \tag{A5}$$

and the average information (AI) matrix

$$\mathrm{AI}(\eta_1, \cdots, \eta_K, \sigma_e^2) = \frac{1}{2}\begin{bmatrix} \mathbf{y}^{\mathrm{T}}\mathbf{PG}_1\mathbf{PG}_1\mathbf{Py} & \cdots & \mathbf{y}^{\mathrm{T}}\mathbf{PG}_1\mathbf{PG}_K\mathbf{Py} & \mathbf{y}^{\mathrm{T}}\mathbf{PG}_1\mathbf{PRPy} \\ \vdots & \ddots & \vdots & \vdots \\ \mathbf{y}^{\mathrm{T}}\mathbf{PG}_K\mathbf{PG}_1\mathbf{Py} & \cdots & \mathbf{y}^{\mathrm{T}}\mathbf{PG}_K\mathbf{PG}_K\mathbf{Py} & \mathbf{y}^{\mathrm{T}}\mathbf{PG}_K\mathbf{PRPy} \\ \mathbf{y}^{\mathrm{T}}\mathbf{PRPG}_1\mathbf{Py} & \cdots & \mathbf{y}^{\mathrm{T}}\mathbf{PRPG}_K\mathbf{Py} & \mathbf{y}^{\mathrm{T}}\mathbf{PRPRPy} \end{bmatrix}. \tag{A6}$$

These two information matrices lead to two slightly different implementations of Newton's method for REML: Fisher-scoring REML and AI-REML (Gilmour, et al., 1995; Johnson and Thompson, 1995). The Fisher-scoring REML employs the following iterative procedure for $\boldsymbol{\theta} = [\eta_1 \ \cdots \ \eta_K \ \sigma_e^2]^{\mathrm{T}}$:

$$\boldsymbol{\theta}_{m+1} = \boldsymbol{\theta}_m + (\mathrm{FI})^{-1}\nabla\ell = \boldsymbol{\theta}_m - \left.\left(\begin{bmatrix} \mathrm{tr}(\mathbf{PG}_1\mathbf{PG}_1) & \cdots & \mathrm{tr}(\mathbf{PG}_1\mathbf{PG}_K) & \mathrm{tr}(\mathbf{PG}_1\mathbf{PR}) \\ \vdots & \ddots & \vdots & \vdots \\ \mathrm{tr}(\mathbf{PG}_K\mathbf{PG}_1) & \cdots & \mathrm{tr}(\mathbf{PG}_K\mathbf{PG}_K) & \mathrm{tr}(\mathbf{PG}_K\mathbf{PR}) \\ \mathrm{tr}(\mathbf{PRPG}_1) & \cdots & \mathrm{tr}(\mathbf{PRPG}_K) & \mathrm{tr}(\mathbf{PRPR}) \end{bmatrix}^{-1}\begin{bmatrix} \mathrm{tr}(\mathbf{PG}_1) - \mathbf{y}^{\mathrm{T}}\mathbf{PG}_1\mathbf{Py} \\ \vdots \\ \mathrm{tr}(\mathbf{PG}_K) - \mathbf{y}^{\mathrm{T}}\mathbf{PG}_K\mathbf{Py} \\ \mathrm{tr}(\mathbf{PR}) - \mathbf{y}^{\mathrm{T}}\mathbf{PRPy} \end{bmatrix}\right)\right|_{\boldsymbol{\theta}=\boldsymbol{\theta}_m}, \tag{A7}$$

where $\boldsymbol{\theta}_m$ represents the values obtained at the m-th iteration. Note that $\mathbf{P}$ needs to be updated using $\boldsymbol{\theta}_m$ at each iteration. It is straightforward to show

$$
\begin{bmatrix} \mathrm{tr}(\mathbf{PG}_1\mathbf{PG}_1) & \cdots & \mathrm{tr}(\mathbf{PG}_1\mathbf{PG}_K) & \mathrm{tr}(\mathbf{PG}_1\mathbf{PR}) \\ \vdots & \ddots & \vdots & \vdots \\ \mathrm{tr}(\mathbf{PG}_K\mathbf{PG}_1) & \cdots & \mathrm{tr}(\mathbf{PG}_K\mathbf{PG}_K) & \mathrm{tr}(\mathbf{PG}_K\mathbf{PR}) \\ \mathrm{tr}(\mathbf{PRPG}_1) & \cdots & \mathrm{tr}(\mathbf{PRPG}_K) & \mathrm{tr}(\mathbf{PRPR}) \end{bmatrix} \begin{bmatrix} \eta_1 \\ \vdots \\ \eta_K \\ \sigma_e^2 \end{bmatrix} = \begin{bmatrix} \mathrm{tr}(\mathbf{PG}_1\mathbf{PV}) \\ \vdots \\ \mathrm{tr}(\mathbf{PG}_K\mathbf{PV}) \\ \mathrm{tr}(\mathbf{PRPV}) \end{bmatrix} = \begin{bmatrix} \mathrm{tr}(\mathbf{PG}_1) \\ \vdots \\ \mathrm{tr}(\mathbf{PG}_K) \\ \mathrm{tr}(\mathbf{PR}) \end{bmatrix}, \tag{A8}
$$

because $\mathbf{PVP} = \mathbf{P}$. Therefore,

$$
\boldsymbol{\theta}_m = \left(\begin{bmatrix} \mathrm{tr}(\mathbf{PG}_1\mathbf{PG}_1) & \cdots & \mathrm{tr}(\mathbf{PG}_1\mathbf{PG}_K) & \mathrm{tr}(\mathbf{PG}_1\mathbf{PR}) \\ \vdots & \ddots & \vdots & \vdots \\ \mathrm{tr}(\mathbf{PG}_K\mathbf{PG}_1) & \cdots & \mathrm{tr}(\mathbf{PG}_K\mathbf{PG}_K) & \mathrm{tr}(\mathbf{PG}_K\mathbf{PR}) \\ \mathrm{tr}(\mathbf{PRPG}_1) & \cdots & \mathrm{tr}(\mathbf{PRPG}_K) & \mathrm{tr}(\mathbf{PRPR}) \end{bmatrix}^{-1} \begin{bmatrix} \mathrm{tr}(\mathbf{PG}_1) \\ \vdots \\ \mathrm{tr}(\mathbf{PG}_K) \\ \mathrm{tr}(\mathbf{PR}) \end{bmatrix} \right) \Bigg|_{\boldsymbol{\theta} = \boldsymbol{\theta}_m} . \tag{A9}
$$

We can use equation (A9) to transform equation (A7) into

$$
\boldsymbol{\theta}_{m+1} = \left(\begin{bmatrix} \mathrm{tr}(\mathbf{PG}_1\mathbf{PG}_1) & \cdots & \mathrm{tr}(\mathbf{PG}_1\mathbf{PG}_K) & \mathrm{tr}(\mathbf{PG}_1\mathbf{PR}) \\ \vdots & \ddots & \vdots & \vdots \\ \mathrm{tr}(\mathbf{PG}_K\mathbf{PG}_1) & \cdots & \mathrm{tr}(\mathbf{PG}_K\mathbf{PG}_K) & \mathrm{tr}(\mathbf{PG}_K\mathbf{PR}) \\ \mathrm{tr}(\mathbf{PRPG}_1) & \cdots & \mathrm{tr}(\mathbf{PRPG}_K) & \mathrm{tr}(\mathbf{PRPR}) \end{bmatrix}^{-1} \begin{bmatrix} \mathbf{y}^{\mathrm{T}}\mathbf{PG}_1\mathbf{Py} \\ \vdots \\ \mathbf{y}^{\mathrm{T}}\mathbf{PG}_K\mathbf{Py} \\ \mathbf{y}^{\mathrm{T}}\mathbf{PRPy} \end{bmatrix} \right) \Bigg|_{\boldsymbol{\theta} = \boldsymbol{\theta}_m} , \tag{A10}
$$

which is identical to equation (A1) in I-MINQUE.

Comparing equation (A5) with (A6) can reveal that AI-REML is more computationally convenient and efficient because it avoids the evaluation of traces in the FI matrix. However, we choose to use Fisher-scoring REML because it often leads to faster convergence (result not shown) and directly reduces to MINQUE with one iteration. The evaluation of traces in the FI matrix is expedited by stochastic trace estimation (see Section 1.4).

1.3. Trust-region dogleg method

In the iterative procedure of I-MINQUE or REML, updating the VC values at the current iteration and then using them to update $\mathbf{V}$ may cause $\mathbf{V}$ to lose positive definiteness in the next iteration, resulting in premature termination. Imposing positive constraints on VC estimates can mitigate this problem, but it compromises the unbiasedness of the estimates. Therefore, to maintain unbiasedness, we cannot apply positive constraints, which may result in frequent convergence failures, especially in models involving many VCs. The convergence problem in REML can be tackled by a trust-region method, as implemented by Loh, et al. (2015) within their software, BOLT. This method optimizes an update step for $\boldsymbol{\theta}$, increasing the REML log-likelihood while ensuring that the step remains within an adaptive trust region. Our implementation of the trust-region method closely mirrors BOLT, except for the step computation strategy. While BOLT

utilizes three optimization routines within NLopt (https://github.com/stevengj/nlopt), we employ Powell's dogleg method (Powell, 1970) in MPH, which integrates Newton's method with steepest descent. Denoting the Newton step and the steepest descent step at an iteration as $\mathbf{p}^{\mathrm{B}}$ and $\mathbf{p}^{\mathrm{U}}$, respectively, the actual update step $\mathbf{p}$ is optimized with respect to s and t ($s > 0$ and $t \geq 0$ and $t + s = 1$, or $s = 0$ and $0 < t \leq 1$) as $\mathbf{p} = s\mathbf{p}^{\mathrm{B}} + t\mathbf{p}^{\mathrm{U}}$, subject to the trust region constraint.

1.4. Stochastic trace estimator

We employ the Girard-Hutchinson trace estimator (Epperly, et al., 2024; Girard, 1989; Hutchinson, 1990) to evaluate the traces in the FI matrix. Let $\mathbf{v}_1, \cdots, \mathbf{v}_S$ denote S identical and independent random vectors of size N, with each vector sampled from $\mathcal{N}(\mathbf{0}, \mathbf{I})$ or the Rademacher distribution $\mathrm{Unif}\{\pm 1\}^N$. The Girard-Hutchinson trace estimator uses the following equation to obtain an unbiased estimator of the trace of a square matrix $\mathbf{A}$:

$$\mathrm{tr}(\mathbf{A}) = \frac{1}{S}\sum_{i=1}^{S} \mathbf{v}_i^{\mathrm{T}} \mathbf{A} \mathbf{v}_i \,. \tag{A11}$$

While the Rademacher distribution can theoretically result in a smaller variance of the trace estimate than the standard normal (Martinsson and Tropp, 2020), there is in practice little difference in their performance. As the number of random vectors (S) increases, both distributions yield estimators with a variance that decreases at a rate of $1/S$. The Girard-Hutchinson trace estimator appears sufficient for REML optimization, despite the availability of improved stochastic trace estimators (Epperly, et al., 2024).

The use of the standard normal distribution in the Girard-Hutchinson trace estimator to evaluate the traces in the gradient mirrors the Monte Carlo algorithm for AI-REML implemented in BOLT (Loh, et al., 2015). Following BOLT, we adopt a default setting of 100 random vectors for VC estimation in MPH. These random vectors are reused across REML iterations. Additionally, we scale the sampling variance-covariance matrix of VC estimates by $1+1/S$, in line with BOLT, to account for the uncertainty imposed by stochastic trace estimation.

Note that we have the following equation for each element (i, j) in the FI matrix:

$$\mathrm{tr}(\mathbf{PG}_i\mathbf{PG}_j) = \mathrm{tr}(\mathbf{PVPG}_i\mathbf{PG}_j) = \mathrm{tr}(\mathbf{L}^{\mathrm{T}}\mathbf{PG}_i\mathbf{PG}_j\mathbf{PL}), \tag{A12}$$

where $\mathbf{V} = \mathbf{LL}^{\mathrm{T}}$ (that is, Cholesky decomposition of $\mathbf{V}$). Our investigation revealed that evaluating $\mathrm{tr}(\mathbf{L}^{\mathrm{T}}\mathbf{PG}_i\mathbf{PG}_j\mathbf{PL})$ by the Girard-Hutchinson trace estimator could result in notably improved numerical stability compared to evaluating $\mathrm{tr}(\mathbf{PG}_i\mathbf{PG}_j)$ (result not shown). Below, we present

the pseudo-code for our implementation of the estimator.

Algorithm ComputeFisherInformation

Input: $\widetilde{\mathbf{Y}} = [\mathbf{v}_1 \cdots \mathbf{v}_S]$ sampled from the standard normal or the Rademacher distribution

Output: The Fisher information (FI) matrix

1. Cholesky decomposition: $\mathbf{V} = \mathbf{L}\mathbf{L}^{\mathrm{T}}$.
2. Compute terms in **P** using **V**'s Cholesky decomposition: $\mathbf{V}^{-1}\mathbf{X}$ and $\mathbf{X}^{\mathrm{T}}\mathbf{V}^{-1}\mathbf{X}$.
3. Compute $\widetilde{\mathbf{Y}}^{*} = \mathbf{L}^{-\mathrm{T}}\widetilde{\mathbf{Y}}$.
4. Compute $\mathbf{PL}\widetilde{\mathbf{Y}} = \widetilde{\mathbf{Y}}^{*} - \mathbf{V}^{-1}\mathbf{X}\left(\mathbf{X}^{\mathrm{T}}\mathbf{V}^{-1}\mathbf{X}\right)^{-1}\mathbf{X}^{\mathrm{T}}\widetilde{\mathbf{Y}}^{*}$.
5. For each VC *i*:
    a. Compute $\mathbf{G}_i\left(\mathbf{PL}\widetilde{\mathbf{Y}}\right)$.
    b. Compute $\mathbf{V}^{-1}\left(\mathbf{G}_i\mathbf{PL}\widetilde{\mathbf{Y}}\right)$.
    c. Compute $\mathbf{PG}_i\mathbf{PL}\widetilde{\mathbf{Y}} = \left(\mathbf{V}^{-1}\mathbf{G}_i\mathbf{PL}\widetilde{\mathbf{Y}}\right) - \mathbf{V}^{-1}\mathbf{X}\left(\mathbf{X}^{\mathrm{T}}\mathbf{V}^{-1}\mathbf{X}\right)^{-1}\mathbf{X}^{\mathrm{T}}\left(\mathbf{V}^{-1}\mathbf{G}_i\mathbf{PL}\widetilde{\mathbf{Y}}\right)$.
6. For each element (*i*, *j*) in the lower triangular part of the FI matrix:
    a. Compute the element-wise product of $\mathbf{G}_i\mathbf{PL}\widetilde{\mathbf{Y}}$ and $\mathbf{PG}_j\mathbf{PL}\widetilde{\mathbf{Y}}$.
    b. Sum all the elements in the product.
    c. FI(*i*, *j*) = the sum divided by *S*.
7. Fill the strictly upper triangular part of FI and return FI.

2. Phenotype simulation

We simulate total genetic values (**g**) by sampling **g** from $\mathcal{N}(\mathbf{0}, \sum_k \mathbf{G}_k\eta_k)$, where η_k represents the k-th variance component. Residuals (**e**) are sampled from $\mathcal{N}(\mathbf{0}, \mathbf{I}\,(1-h^2)\sum_k \eta_k/h^2)$, where h^2 represents heritability. Phenotypes (**y**) are then obtained by $\mathbf{y} = \mathbf{g} + \mathbf{e}$. This phenotype simulation process has been implemented in MPH.

Supplementary Figures

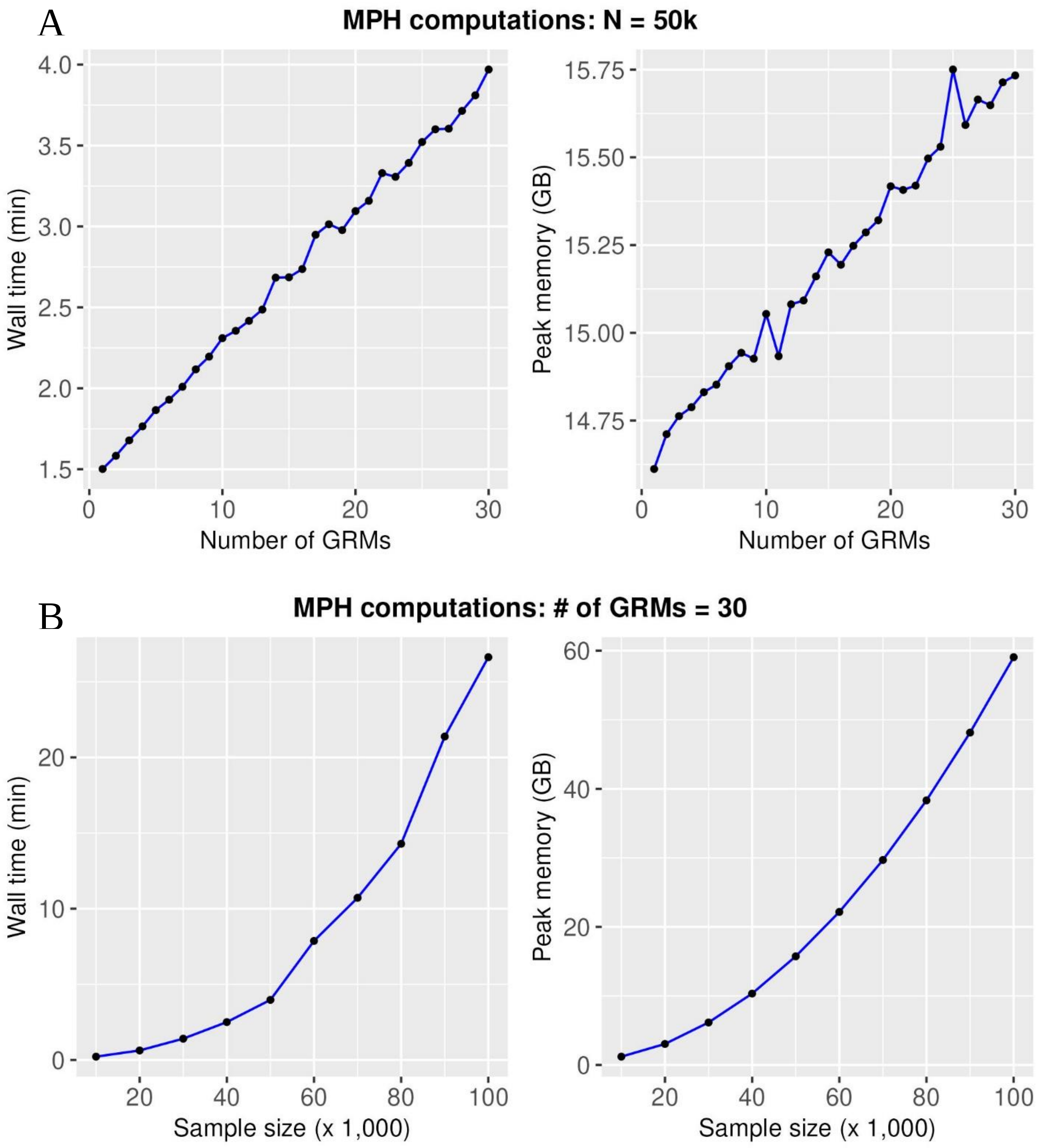

Supplementary Figure S1. Computational performance of MPH REML. A. Computational performance of MPH REML for a fixed sample size of 50,000 with varying numbers of genomic relationship matrices (GRMs). B. Computational performance of MPH REML for a fixed set of 30 GRMs across a range of sample sizes. The wall time encompasses all operations from reading

input files (including GRMs and phenotypes) to solving REML equations and writing output files. However, it does not include the computation of GRMs. The computations were performed using 14 Intel Xeon Gold 6258R CPU cores.

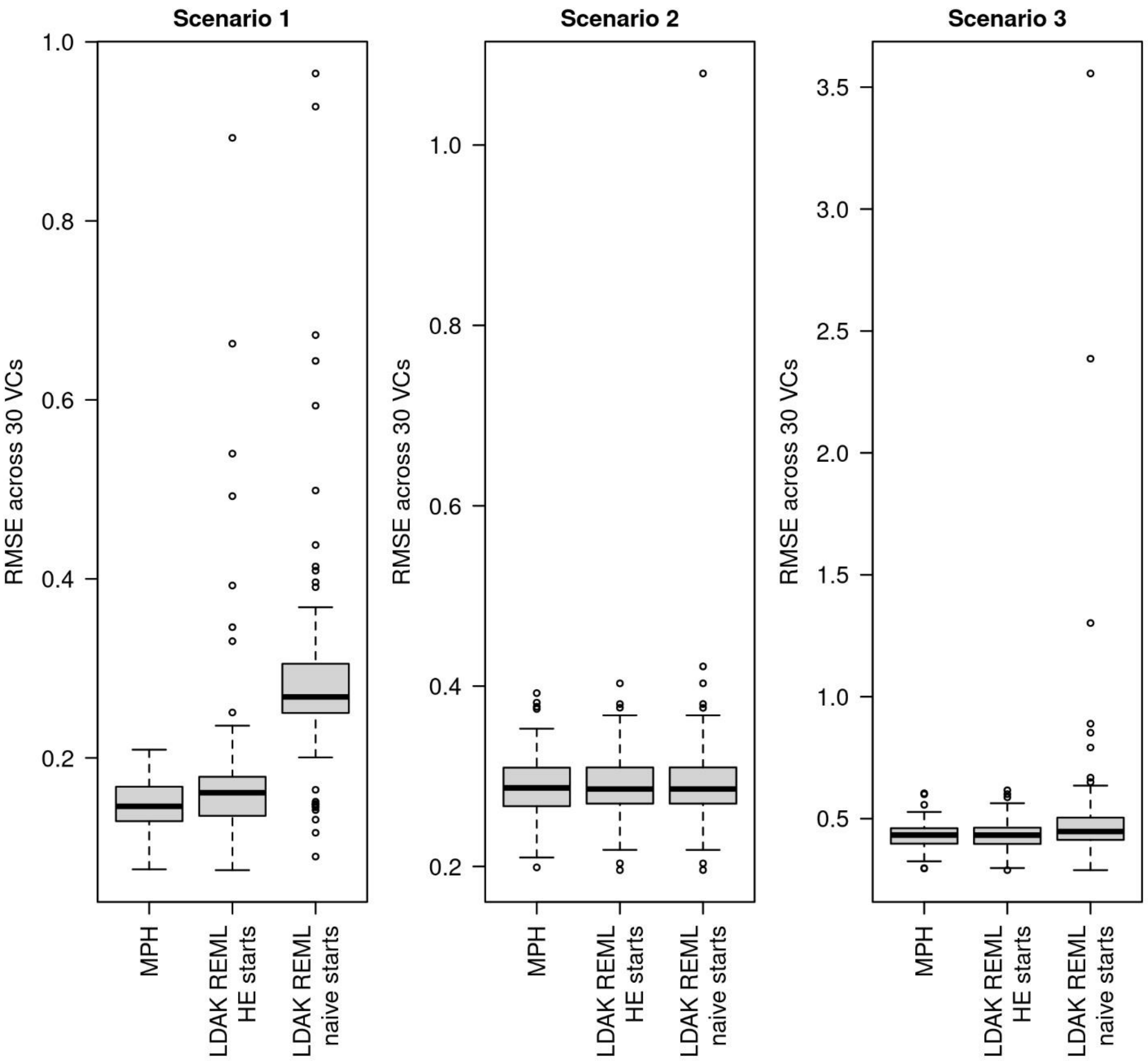

Supplementary Figure S2. Root mean square error (RMSE) of variance component (VC) estimates. The RMSE was calculated across 30 chromosomes for each of the 100 replicates under each scenario. Scenario 1: true VC = 1 for chromosomes 1-15 and VC = 0 for 16-30. Scenario 2: true VC = 1 for all 30 chromosomes. Scenario 3: true VC = 1 for chromosomes 1-15 and VC = 2 for 16-30.

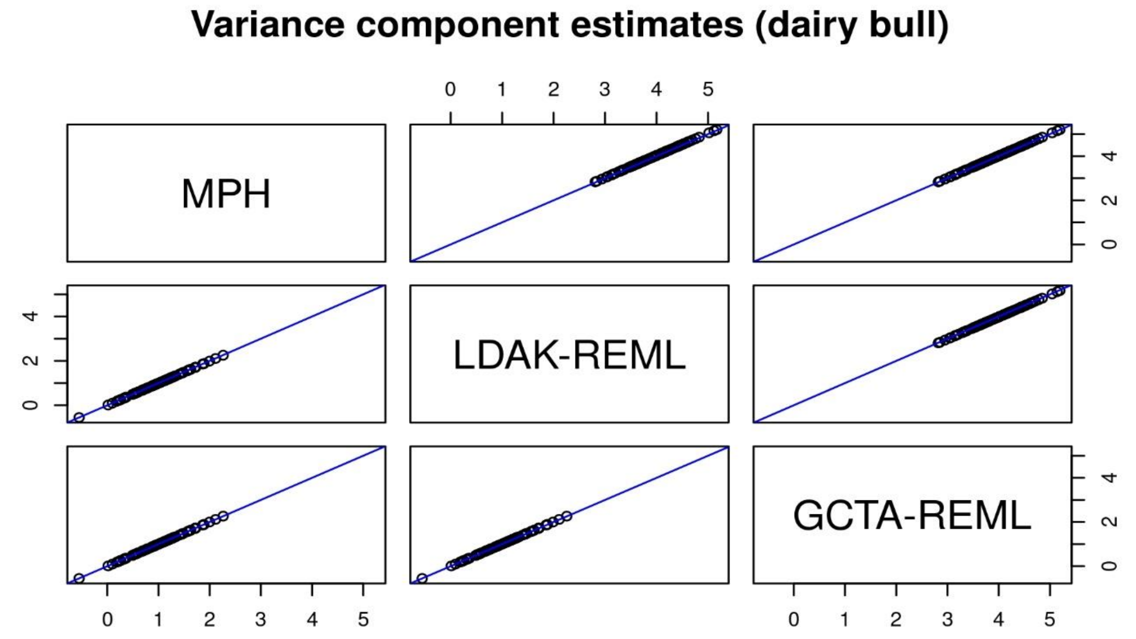

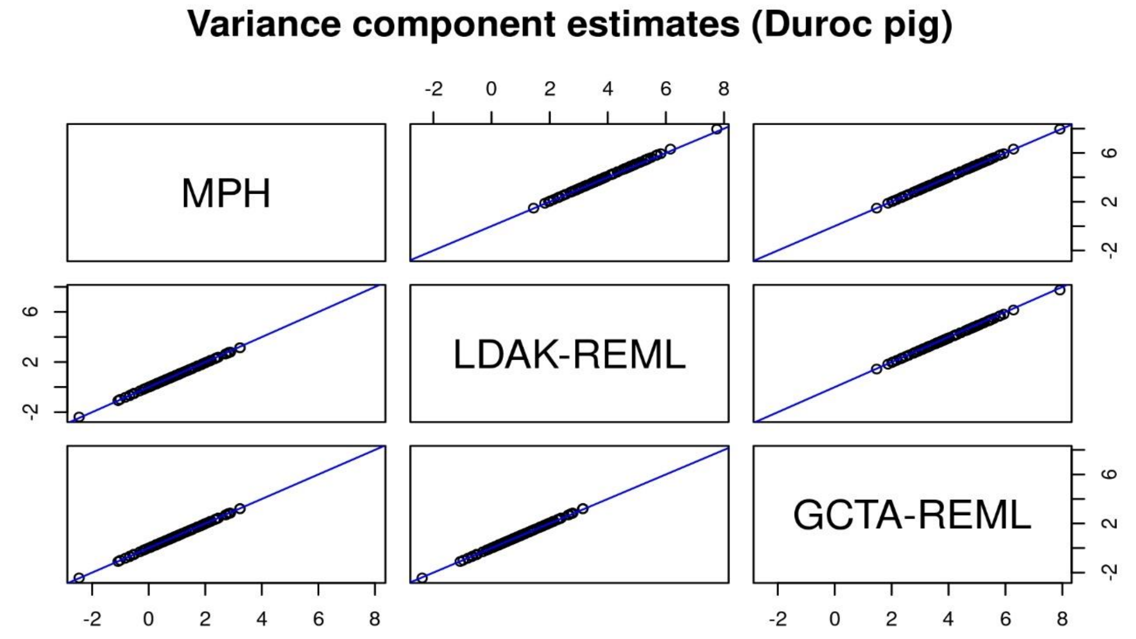

Supplementary Figure S3. Variance component (VC) estimates of three REML methods for simulated traits based on real genotypes of dairy bulls and Duroc pigs. Each panel below the diagonal shows the estimates of 100 replicate traits for VC 1 (true value = 1), while each panel above the diagonal shows the estimates for VC 2 (true value = 4). Blue lines represent *y*=*x*. LDAK-REML used average information, while MPH and GCTA-REML used Fisher's scoring methods.

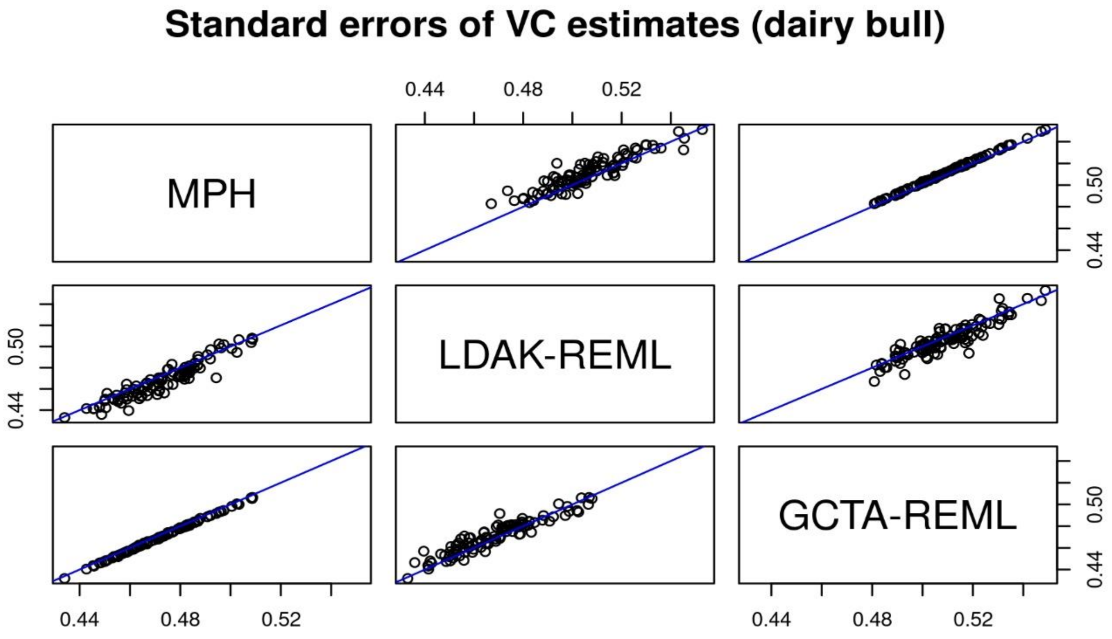

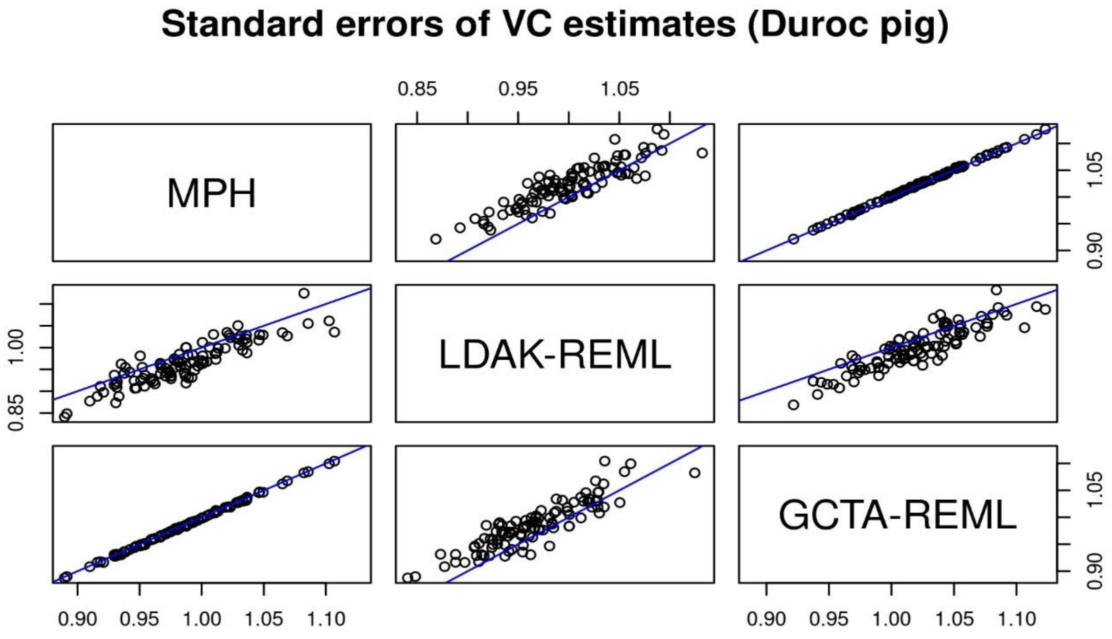

Supplementary Figure S4. Analytical standard errors (SEs) of variance component (VC) estimates of three REML methods for simulated traits based on real genotypes of dairy bulls and Duroc pigs. Each panel below the diagonal shows the SEs of 100 replicate traits for VC 1, while each panel above the diagonal shows the SEs for VC 2. Blue lines represent *y*=*x*. LDAK-REML used average information, while MPH and GCTA-REML used Fisher's scoring method.

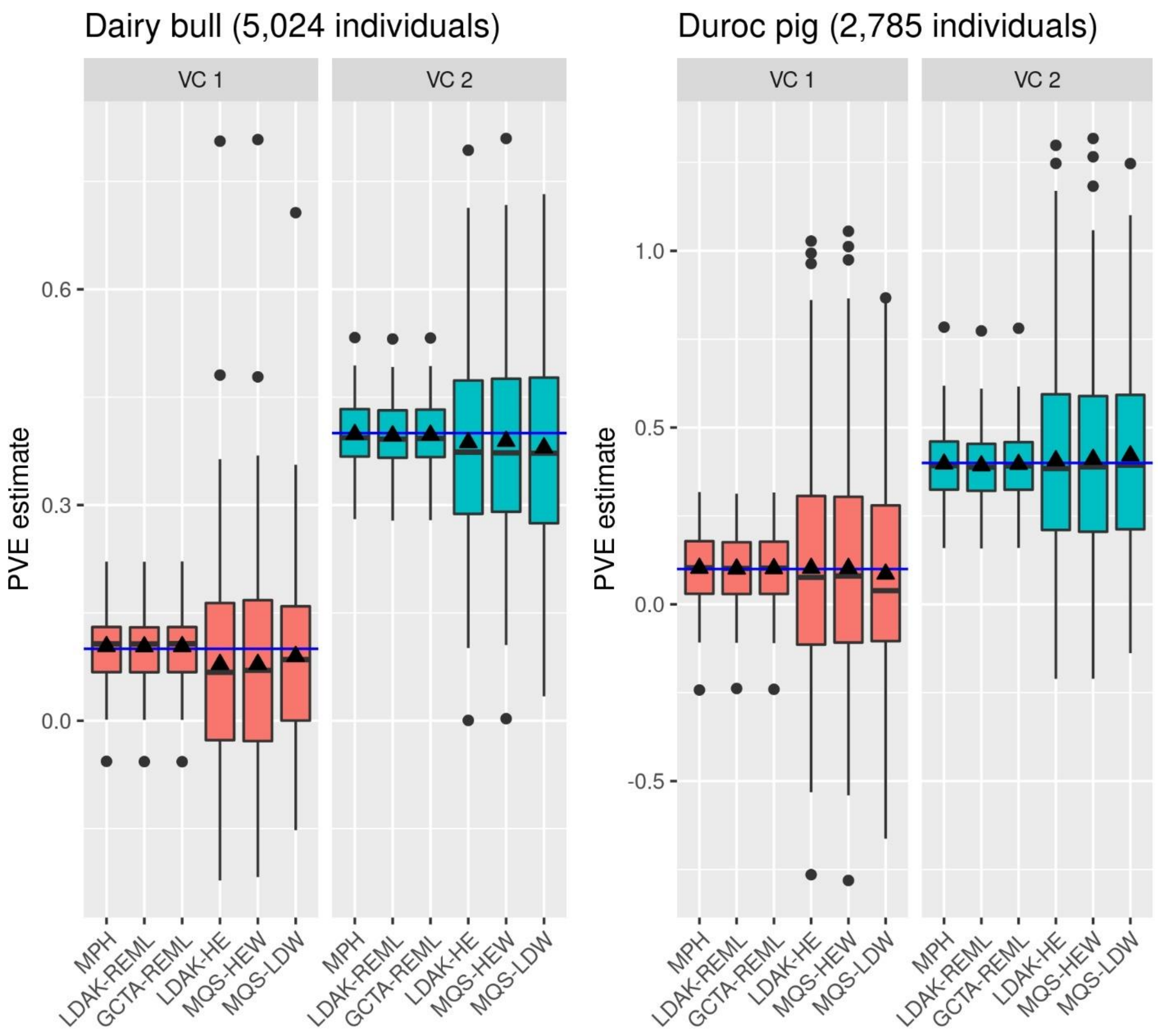

Supplementary Figure S5. Comparison of the proportions of variance explained (PVE) by two genetic components estimated by REML, Haseman-Elston (HE) regression, and MQS for simulated traits. These traits were simulated using real genotypes of dairy bulls and Duroc pigs. Blue lines represent true PVE values (0.1 and 0.4). Black triangles represent mean values across 100 replicates. LDAK-REML used average information, while MPH and GCTA-REML used Fisher's scoring method. LDAK-HE represents the HE regression in LDAK. In MQS (-HEW and -LDW), individual-level genotypes from the entire population were used as the reference. For the MQS-LDW -wcat option, a SNP's LD score for each VC was computed as the sum of observed squared correlations between the SNP and genome-wide SNPs belonging to that VC.

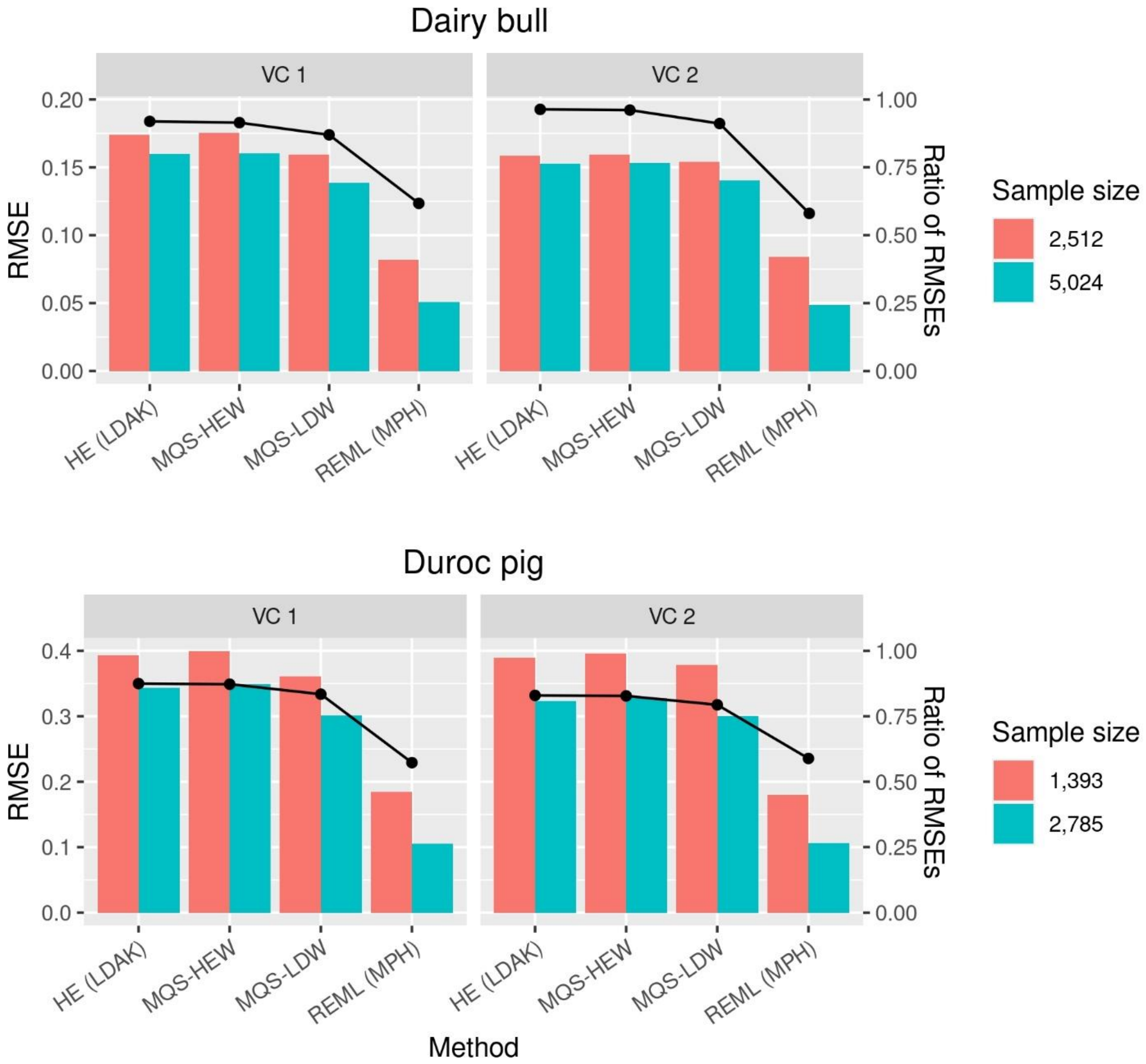

Supplementary Figure S6. Root mean square error (RMSE) of estimates of the proportion of variance explained (PVE) by each genetic component across 100 simulated replicate traits. These traits were simulated using real genotypes of dairy bulls and Duroc pigs. VCs 1 and 2 have true PVE values of 0.1 and 0.4, respectively. The bars represent RMSE values, while the black lines and points represent the ratio of RMSEs obtained from the full data and half the data. HE (LDAK) and REML (MPH) denote the HE regression in LDAK and REML in MPH, respectively. In MQS (-HEW and -LDW), individual-level genotypes from the entire population were used as the reference. For the MQS-LDW -wcat option, a SNP's LD score for each VC was computed as the sum of observed squared correlations between the SNP and genome-wide SNPs belonging to that VC.

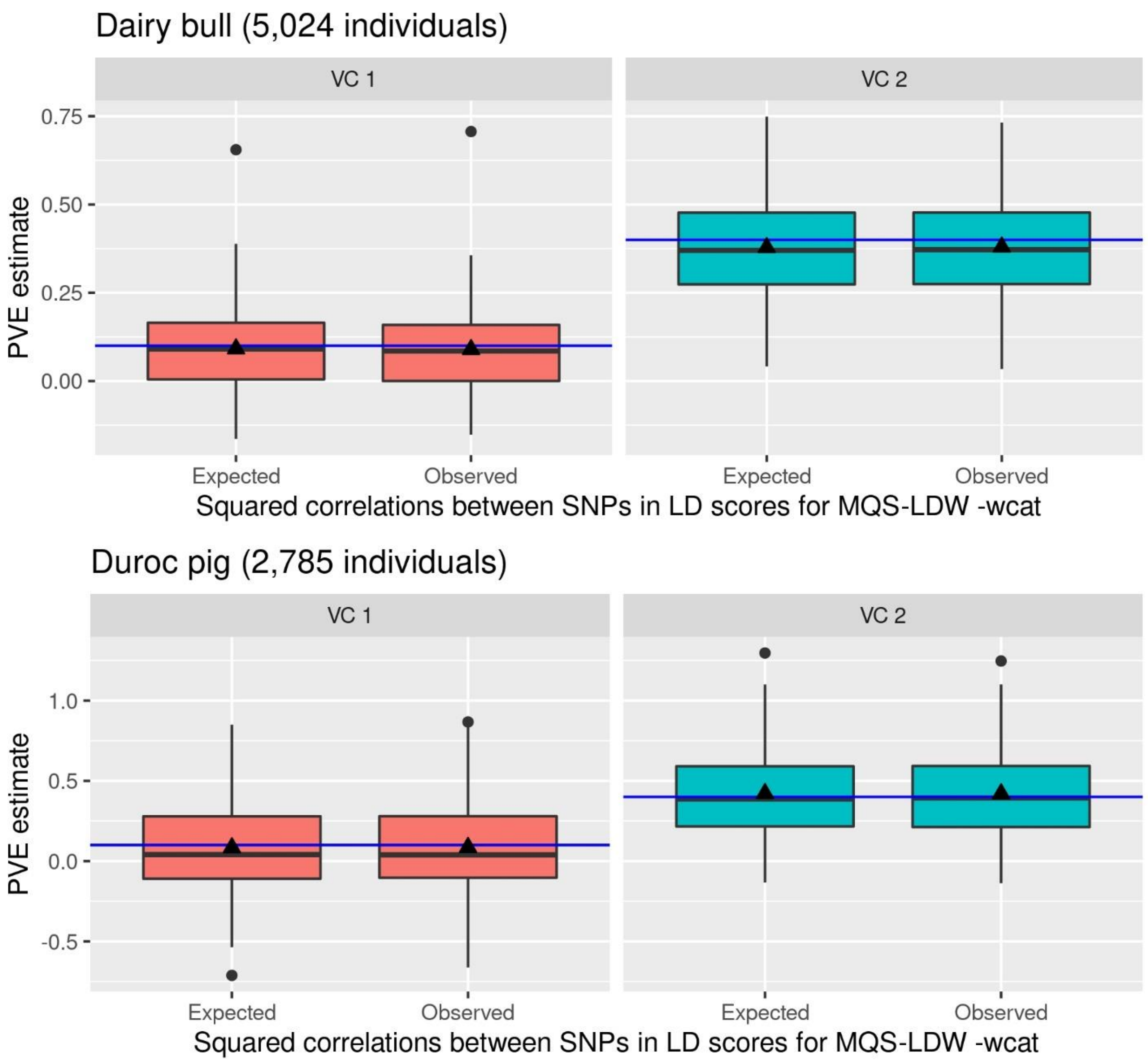

Supplementary Figure S7. Comparison of the proportions of variance explained (PVE) for simulated traits by two genetic components, estimated using MQS-LDW with two different methods for calculating LD scores. The LD scores were calculated using individual-level genotypes from the entire population and used for the MQS-LDW -wcat option. The terms "Expected" and "Observed" refer to the expected and observed squared correlations, respectively, between SNPs.

Supplementary Tables

Supplementary Table S1. The estimated proportion of variance explained (PVE) by each of two genetic components for milk yield (MY), milk fat percentage (MFP), and somatic cell score (SCS) in the dairy bull dataset

Traits	VC	MPH		LDAK-REML		GCTA-REML	
		Estimate	SE	Estimate	SE	Estimate	SE
MY	1	0.384	0.042	0.383	0.043	0.383	0.041
	2	0.425	0.041	0.424	0.043	0.425	0.041
MFP	1	0.415	0.035	0.414	0.038	0.414	0.035
	2	0.508	0.035	0.509	0.038	0.509	0.035
SCS	1	0.432	0.042	0.432	0.041	0.432	0.041
	2	0.375	0.042	0.374	0.041	0.374	0.041

The REML analysis used two genomic relationship matrices (GRMs) constructed from equally split SNP sets. LDAK-REML used average information, while MPH and GCTA-REML used Fisher's scoring method. The combined PVE for the two genetic VCs in each trait is high, attributed to the use of pseudo-phenotypes (i.e., estimated breeding values). Sample size = 5,024.

Supplementary Table S2. The estimated proportion of variance explained (PVE) by each of two genetic components for back fat (BF), loin muscle depth (LMD), and total teat number (TTN) in the Duroc pig dataset

Traits	VC	MPH		LDAK-REML		GCTA-REML	
		Estimate	SE	Estimate	SE	Estimate	SE
BF	1	0.197	0.096	0.191	0.094	0.194	0.096
	2	0.087	0.096	0.086	0.093	0.088	0.095
LMD	1	0.285	0.097	0.278	0.096	0.282	0.097
	2	0.025	0.095	0.026	0.093	0.026	0.095
TTN	1	0.180	0.097	0.175	0.094	0.177	0.097
	2	0.130	0.097	0.128	0.093	0.129	0.096

The REML analysis used two genomic relationship matrices (GRMs) constructed from equally split SNP sets. LDAK-REML used average information, while MPH and GCTA-REML used Fisher's scoring method. Sample size = 2,785.

References

Brown, K.G. Asymptotic Behavior of Minque-Type Estimators of Variance Components. *The Annals of Statistics* 1976;4(4):746-754, 749.

Epperly, E.N., Tropp, J.A. and Webber, R.J. Xtrace: Making the most of every sample in stochastic trace estimation. *SIAM Journal on Matrix Analysis and Applications* 2024;45(1):1-23.

Gilmour, A.R., Thompson, R. and Cullis, B.R. Average Information REML: An Efficient Algorithm for Variance Parameter Estimation in Linear Mixed Models. *Biometrics* 1995;51(4):1440-1450.

Girard, A. A fast 'Monte-Carlo cross-validation' procedure for large least squares problems with noisy data. *Numerische Mathematik* 1989;56(1):1-23.

Hutchinson, M.F. A stochastic estimator of the trace of the influence matrix for laplacian smoothing splines. *Communications in Statistics - Simulation and Computation* 1990;19(2):433-450.

Johnson, D.L. and Thompson, R. Restricted Maximum Likelihood Estimation of Variance Components for Univariate Animal Models Using Sparse Matrix Techniques and Average Information. *Journal of Dairy Science* 1995;78(2):449-456.

Loh, P.R.*, et al.* Contrasting genetic architectures of schizophrenia and other complex diseases using fast variance-components analysis. *Nat Genet* 2015;47(12):1385-1392.

Martinsson, P.-G. and Tropp, J.A. Randomized numerical linear algebra: Foundations and algorithms. *Acta Numerica* 2020;29:403-572.

Pazokitoroudi, A.*, et al.* Efficient variance components analysis across millions of genomes. *Nat Commun* 2020;11(1):4020.

Powell, M.J. A hybrid method for nonlinear equations, numerical methods for nonlinear algebraic equations. *Numerical methods for non-linear algebraic equations* 1970:87-114.

Rao, C.R. Estimation of variance and covariance components—MINQUE theory. *Journal of multivariate analysis* 1971;1(3):257-275.

Rao, C.R. Minimum variance quadratic unbiased estimation of variance components. *Journal of Multivariate Analysis* 1971;1(4):445-456.

Rao, C.R. Estimation of variance and covariance components in linear models. *Journal of the American Statistical Association* 1972;67(337):112-115.

Swallow, W.H. and Monahan, J.F. Monte Carlo Comparison of ANOVA, MIVQUE, REML, and ML Estimators of Variance Components. *Technometrics* 1984;26(1):47-57.
